# Pharmacological activation of transient receptor potential vanilloid 4 promotes triggering of the swallowing reflex in rats

**DOI:** 10.3389/fncel.2023.1149793

**Published:** 2023-02-22

**Authors:** Mohammad Zakir Hossain, Hiroshi Ando, Shumpei Unno, Rita Rani Roy, Junichi Kitagawa

**Affiliations:** ^1^Department of Oral Physiology, School of Dentistry, Matsumoto Dental University, Shiojiri, Japan; ^2^Department of Biology, School of Dentistry, Matsumoto Dental University, Shiojiri, Japan

**Keywords:** transient receptor potential vanilloid 4, swallowing reflex, GSK1016790A, superior laryngeal nerve-afferents, nodose–petrosal–jugular ganglionic complex

## Abstract

The swallowing reflex is an essential physiological reflex that allows food or liquid to pass into the esophagus from the oral cavity. Delayed triggering of this reflex is a significant health problem in patients with oropharyngeal dysphagia for which no pharmacological treatments exist. Transient receptor potential channels have recently been discovered as potential targets to facilitate triggering of the swallowing reflex. However, the ability of transient receptor potential vanilloid 4 (TRPV4) to trigger the swallowing reflex has not been studied. Here, we demonstrate the involvement of TRPV4 in triggering the swallowing reflex in rats. TRPV4 immunoreactive nerve fibers were observed in the superior laryngeal nerve (SLN)-innervated swallowing-related regions. Retrograde tracing with fluorogold revealed localization of TRPV4 on approximately 25% of SLN-afferent neurons in the nodose–petrosal–jugular ganglionic complex. Among them, approximately 49% were large, 35% medium, and 15% small-sized SLN-afferent neurons. Topical application of a TRPV4 agonist (GSK1016790A) to the SLN-innervated regions dose-dependently facilitated triggering of the swallowing reflex, with the highest number of reflexes triggered at a concentration of 250 μM. The number of agonist-induced swallowing reflexes was significantly reduced by prior topical application of a TRPV4 antagonist. These findings indicate that TRPV4 is expressed on sensory nerves innervating the swallowing-related regions, and that its activation by an agonist can facilitate swallowing. TRPV4 is a potential pharmacological target for the management of oropharyngeal dysphagia.

## 1. Introduction

The swallowing reflex is a vital reflex triggered when a food bolus passes from the oral cavity to the esophagus (Jean, 2001; Yamamura et al., 2010; Hossain et al., 2020a). The reflex also protects the airway by preventing entry of food or liquid (Jean, 2001; Yamamura et al., 2010; Nishino, 2013). Difficulty or delay in triggering this reflex is a significant problem in patients with oropharyngeal dysphagia associated with neurological disorders (e.g., stroke) that can lead to the entry of food particles into the lungs and aspiration pneumonia (Cabre et al., 2009; Clavé and Shaker, 2015). Delay in triggering this reflex is also a major health problem in the aged population (Cabre et al., 2009; Carrión et al., 2015). Despite its clinical significance, there is no established pharmacological treatment for oropharyngeal dysphagia (Hossain et al., 2020a; Cheng et al., 2022). Recently, transient receptor potential (TRP) channels have been targeted to develop a pharmacological treatment strategy for oropharyngeal dysphagia (Hossain et al., 2020a; Tomsen et al., 2022). We have previously observed that activation of TRP vanilloid 1 (TRPV1), TRP ankyrin 1 (TRPA1), and TRP melastatin 8 (TRPM8) channels, which are present in the peripheral swallowing-related regions, trigger the swallowing reflex (Hossain et al., 2018, 2022). Those observations prompted us to examine the involvement of transient receptor potential vanilloid 4 (TRPV4) in triggering the reflex. TRPV4 is a non-selective cation channel that is widely expressed in various body structures. It serves as a polymodal receptor to various stimuli, including chemical stimuli (White et al., 2016; Rosenbaum et al., 2020). Various agonists, including GSK1016790A, activate TRPV4 (White et al., 2016; Rosenbaum et al., 2020). To our knowledge, no study has examined the involvement of TRPV4 in triggering the swallowing reflex. Understanding its involvement in triggering the swallowing reflex may help to develop a pharmacological strategy for targeting this channel to help manage oropharyngeal dysphagia.

The superior laryngeal nerve (SLN) (a branch of the vagus nerve) plays an important role in triggering the swallowing reflex (Jean, 2001; Yamamura et al., 2010; Hossain et al., 2020a). We and others have reported that chemical, mechanical or electrical stimulation of SLN-innervated swallowing-related regions (laryngopharynx and associated laryngeal regions) or direct electrical stimulation to the SLN readily triggers the swallowing reflex. This indicates the importance of these regions in triggering the swallowing reflex (Jean, 2001; Yamamura et al., 2010; Mostafeezur et al., 2012; Hossain et al., 2020a). These regions are highly innervated and contain various receptors (Bradley, 2000; Yoshida et al., 2000). The sensory information from these regions is carried through the SLN-afferents to the central pattern generator for swallowing (sCPG) located in the brainstem (Jean, 2001; Yamamura et al., 2010; Hossain et al., 2020a). The cell bodies of SLN-afferents are situated in a ganglionic complex named the nodose–petrosal–jugular ganglionic complex (NPJc) (Yoshida et al., 2000; Wank and Neuhuber, 2001).

This study aimed to understand whether TRPV4 is present in SLN-afferent neurons and whether it functions to trigger the swallowing reflex.

## 2. Materials and methods

Experiments were conducted using 28 male Sprague Dawley rats weighing approximately 250–400 g (immunohistochemistry, *n* = 7; PCR, *n* = 2; swallowing reflex, *n* = 19). The experimental procedures were approved by Matsumoto Dental University Intramural Animal Care and Veterinary Science Committee (Ref. No. 394). We made every effort to minimize animal suffering and the number of animals used. We adhered to the guidelines of the National Centre for the Replacement, Refinement, and Reduction of Animals in Research, ARRIVE (Animal Research: Reporting of *In Vivo* Experiments).

### 2.1. Immunohistochemical analysis of SLN-innervated swallowing-related regions

Deeply anesthetized rats were transcardially perfused with saline and then 4% paraformaldehyde. The swallowing-related regions were dissected and fixed in 4% paraformaldehyde for 24 h. For cryoprotection, specimens were placed in 30% sucrose until they sank. After embedding in Tissue-Tek O.C.T Compound (Sakura Finetek, Tokyo, Japan), samples were sectioned in the sagittal plane (thickness, 10 or 50 μm) and sections mounted on glass slides. For non-specific blocking, sections were incubated with 5% normal goat serum in 0.01 M PBS containing 0.3% Triton X-100 for 1 hour. Sections were then incubated with rabbit monoclonal anti-TRPV4 (1:100; Cat# ab259361; Abcam, Cambridge, UK) and mouse monoclonal anti-protein gene product (PGP) 9.5 (1:200; Cat# ab8189; Abcam) antibodies at room temperature overnight, and then with appropriate secondary antibodies (Alexa Fluor 488; Cat# A-11029; and Alexa Fluor 594; Cat# A-11037; Thermo Fisher Scientific, Waltham, MA, USA) for 2 hours at room temperature. Sections were then incubated with 4’,6-diamidino-2-phenylindole (DAPI) for 10 min to detect cell nuclei. Sections were then coverslipped with a mounting medium (PermaFluor; Thermo Fisher Scientific) and examined using fluorescence microscopy (BZ-X700; Keyence Corp., Osaka, Japan).

### 2.2. Reverse transcription polymerase chain reaction (RT-PCR)

Total ribonucleic acid (RNA) was extracted from the NPJc and trigeminal ganglia (TG) using a NucleoSpin^®^ RNA kit (Macherey-Nagel; Takara Bio Inc., Shiga, Japan). cDNA was synthesized using SuperScript IV (VILO Master Mix with ezDNase™, Thermo Fisher Scientific). PCR was performed with specific primer sets using TaKaRa Ex Taq^®^ (Takara Bio Inc., Shiga, Japan). The sequences of primers were: *TRPV4*, forward 5′-CTTTACTTCACCCGTGGGCT-3′, reverse 5′-CAGTTGCTCTGGTCCTCGTT-3′ (product size 188 bp); β*-actin*, forward 5′-AGACTTCGAGCAAGAGATGG-3′, reverse 5′-AGGAAGGAAGGCTGGAAGAG-3′ (product size 138 bp). β*-actin* was used as a reference gene. PCR products were evaluated by 2% agarose gel electrophoresis in 0.5% Tris-borate-EDTA buffer and visualized after staining with ethidium bromide solution.

### 2.3. Immunohistochemical analysis of the NPJc

Fluorogold (FG) was used to retrogradely trace SLN-afferent neurons in the NPJc. Under pentobarbital anesthesia, the right-sided SLN was isolated from its surrounding tissues after a midline incision in the ventral surface of the neck and transected at its entry to the trachea. The cut end of the nerve was then inserted into a small tube filled with 4% FG. Silicone and cyanoacrylate glue were used to seal the tube ends into which the nerve was inserted. The tube containing the nerve and FG was fixed to the surrounding tissues using the glue, and the incision was sutured. Five to seven days after FG incorporation, deeply anesthetized rats were transcardially perfused with saline followed by 4% paraformaldehyde. The right-sided NPJc was excised and immersed in 4% paraformaldehyde and then in 30% sucrose for cryoprotection until it sank. After embedding in Tissue-Tek O.C.T Compound (Sakura Finetek), specimens were sectioned (thickness, 16 μm) and sections mounted on glass slides. For non-specific blocking, sections were incubated in 0.01 M PBS containing 5% normal goat serum and 0.3% Triton X-100 for 30 min. Sections were then incubated with rabbit monoclonal anti-TRPV4 (1:300; Cat# ab259361; Abcam) at room temperature overnight, and then with a secondary antibody (Alexa Fluor 594; Cat# A-11037; Thermo Fisher Scientific). Sections were then coverslipped and viewed by fluorescence microscopy (BZ-X700; Keyence Corp.). ImageJ software (NIH, Bethesda, MD, USA) was used to count immunoreactive cells. The sections showing the highest number of TRPV4-immunoreactive cells were used for counting. Six sections were chosen from each rat (two sections/ganglion). ImageJ software was used to measure the cell body area of neurons expressing TRPV4 and FG. A cell area >800 μm^2^ was considered large, 400–800 μm^2^ was considered medium, and <400 μm^2^ was considered small (Ichikawa et al., 2005; Sasaki et al., 2013; Table 1).

**TABLE 1:** Cell size distribution of FG-stained TRPV4 immunoreactive neurons in the NPJc.

	Small (<400 μm^2^)	Medium (400–800 μm^2^)	Large (>800 μm^2^)
NG	6.7% (10/150)	34.7% (52/150)	58.7% (88/150)
PG	51.9% (14/27)	29.6% (8/27)	18.5% (5/27)
JG	40.0% (6/15)	53.3% (8/15)	6.7% (1/15)
NPJc	15.6% (30/192)	35.4% (68/192)	49.0% (94/192)

All data were obtained from 30 sections (two sections/ganglion/rat). *n* = 5. The numbers within parenthesis indicate the numbers of neurons analyzed.

Transient receptor potential vanilloid 4 is localized in TG (Denadai-Souza et al., 2012; Chen et al., 2013, 2014, 2020); therefore, we used TG sections as a positive control for the anti-TRPV4 antibody (Supplementary Figure 1). As a negative control, the sections were incubated with a universal negative control reagent (Cat # ADI-950-231-0025; Enzo Life Sciences, Inc., Farmingdale, NY) instead of the primary antibody (Supplementary Figure 1).

### 2.4. Surgery for recording the swallowing reflex

Following urethane anesthesia (1.0–1.5 g/kg, administered intraperitoneally), rats were placed supine and a midline incision was made in the ventral surface of the neck to allow isolation of the trachea. Respiration was maintained using a custom-made cannula inserted into the trachea toward the lungs. A window to deliver solutions was created by surgically removing a small portion of ventral trachea. The bilateral SLNs were left intact but the pharyngeal (IX-ph) and lingual (IX-li) branches of the glossopharyngeal, pharyngeal (X-ph), and recurrent laryngeal (RLN) branches of the vagus nerve were transected bilaterally to avoid the influence of non-SLNs in triggering the swallowing reflex.

### 2.5. Swallowing reflex recording

Electromyogram (EMG) activity of the mylohyoid muscle was recorded to identify the swallowing reflex (Hossain et al., 2018, 2020b,^2022^). The mylohyoid muscle and other infra- and supra-hyoid muscles are activated during the triggering of the swallowing reflex (Jean, 2001; Yamamura et al., 2010). High amplitude EMG activity was observed in the mylohyoid muscle during the triggering of the swallowing reflex. A high amplitude activity event in the EMG signal corresponded to one swallowing reflex. Therefore, triggered swallowing reflexes were identified and counted using high-amplitude EMG of the mylohyoid muscle and by visual checking of laryngeal movements associated with reflex triggering (Hossain et al., 2018, 2020b,^2022^). The examiners used a loudspeaker connected to the EMG signal to facilitate observation of mylohyoid muscle firing. Bipolar fine wire electrodes (stainless-steel and urethane-coated; Unique Medical Co., Ltd., Tokyo, Japan) were used to record EMG activity from the mylohyoid muscle during swallowing. A data acquisition system (Power 1401; Cambridge Electronic Design Ltd., Cambridge, UK) was used to amplify and digitize the EMG signals. The data were stored on a hard drive for subsequent analysis.

### 2.6. Stimulating solutions

The stimulating solutions were saline (NaCl 0.9%, Otsuka Pharmaceutical Co. Ltd. Tokyo, Japan) and GSK1016790A (Tocris Bioscience, Bristol, UK; 1, 10, 50, 100, 250, and 500 μM) and vehicle (vehicle for the highest concentration of GSK1016790A). Desired concentrations of GSK1016790A were prepared by diluting a GSK1016790A stock solution (15 mM, prepared by dissolving in a mixture of 5% DMSO and 5% Tween 80) in saline. In pilot studies, we determined the concentrations of GSK1016790A that triggered a considerable number of swallowing reflexes. A syringe with a 21-gauge needle (blunt tip) was used to deliver the solutions to the SLN-innervated swallowing-related regions. The volume of the delivered solutions was 50 μl. After delivery, triggered reflexes were recorded for 20 s. The time interval between deliveries of different solutions was 2–3 min. During this interval, the delivered solutions were aspirated, and the regions were thoroughly washed 2–3 times with saline. Small pointed pieces of tissue paper were used to absorb the remaining saline from the areas. The solutions were delivered at room temperature (22–24°C).

### 2.7. TRPV4 antagonist

RN-9893 (Tocris Bioscience, Bristol, UK) was used as a TRPV4 antagonist. The efficacy of RN-9893 as a TRPV4 antagonist was previously validated (Wei et al., 2015; Ahn et al., 2020; Al-Shammari et al., 2020). RN-9893 was prepared by dissolving in 1% DMSO (Sigma-Aldrich, St. Louis, MO) and 1% Tween 80 (Sigma-Aldrich, St. Louis, MO, USA) followed by dilution in saline. The DMSO/Tween 80/saline solution was used as vehicle. The TRPV4 antagonist or vehicle was instilled into the SLN-innervated swallowing-related region for 15 min and then aspirated before applying GSK1016790A. Pilot experiments determined the lowest effective concentration of RN-9893 that reduced the 250 μM GSK1016790A-induced swallowing reflexes to ≤50% was 10 mM. Therefore, we used 10 mM RN-9893 to test the effect of a TRPV4 antagonist on GSK1016790A-induced swallowing reflexes.

### 2.8. Data and statistical analysis

The triggered swallowing reflexes were counted for 20 s after applying the stimulating solutions. Additionally, the average interval between the triggered swallowing reflexes was calculated from the reflexes evoked within 10 s following solution delivery. For calculating the swallowing interval, the initial 10 s was chosen because GSK1016790A-induced shortening of the interval was most prominent within this time. The interval between the start of high-amplitude EMG firing for one swallowing reflex and the beginning of high-amplitude EMG firing for the subsequent reflex was used as the interval between the reflexes (Hossain et al., 2020b,^2022^).

For statistical analysis, data were checked by normality and equal variance tests to determine whether to run parametric or non-parametric tests. When both normality and equal variance tests passed, we used parametric tests; otherwise, we used non-parametric tests. Previous studies using a small number of animals also performed similar procedures to determine appropriate statistical tests (Tsujimura et al., 2019; Hossain et al., 2022). The number of the swallowing reflexes triggered by different concentrations of GSK1016790A and the number and intervals of the reflexes with and without prior application of the TRPV4 antagonist or vehicle were compared using one-way repeated measures analysis of variance (ANOVA) followed by Tukey’s test. The intervals of the swallowing reflexes triggered by different concentrations of GSK1016790A were compared using Friedman repeated measures ANOVA on ranks followed by Tukey’s test. The numbers of GSK1016790A-triggered swallowing reflexes with and without prior application of lidocaine or transection of SLNs were compared using a paired *t*-test. Differences were considered significant at *P* < 0.05. The data are shown as the mean ± SEM (standard error of the mean). Sigmaplot software was used for statistical analyses (Sigmaplot 14.0; Systat Software Inc., San Jose, CA, USA). GraphPad Prism Software v9.0 (San Diego, CA, USA) was used to create the column graphs with individual data points.

## 3. Results

### 3.1. TRPV4 is expressed in swallowing-related regions

**FIGURE 1:**
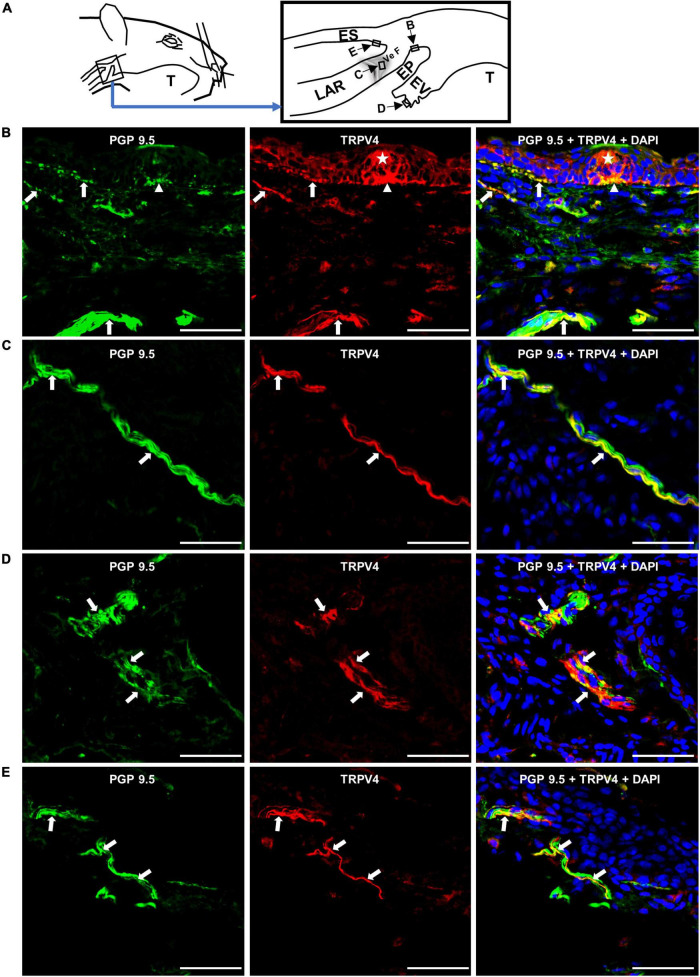
Nerve fibers in the swallowing-related regions express TRPV4. **(A)** Schematic of the SLN-innervated laryngopharyngeal and associated laryngeal regions. Rectangles with arrows and letters show the areas where the photomicrographs were taken. Photomicrographs of TRPV4 localization in the **(B)** epiglottis (EP), **(C)** vestibular fold (Ve F), **(D)** epiglottic vallecula (EV), and **(E)** cervical esophagus (ES). Arrows show examples of TRPV4 on PGP 9.5-expressing nerve fibers in different areas of the swallowing-related regions. The asterisk indicates a taste bud-like structure. Arrowhead indicates a subgemmal neurogenous plaque to the taste bud-like structure expressing TRPV4. Scale bars = 50 μm. LAR, larynx; T, tongue.

We examined whether TRPV4 is localized on nerve fibers in SLN-innervated swallowing-related regions. Nerve fibers were detected using a nerve-fiber marker, PGP 9.5. We observed TRPV4 on some PGP 9.5-expressing nerve fibers (both thin and thick) in different SLN-innervated swallowing-related regions (Figure 1). We also observed TRPV4 in some cells of taste bud-like structures (Figure 1B), epithelial cells in arytenoids and vestibular folds, endothelial-like cells in blood vessels, and cells in seromucous glands (not shown in the figure).

### 3.2. TRPV4 is expressed on SLN-afferent neurons in the NPJc

**FIGURE 2:**
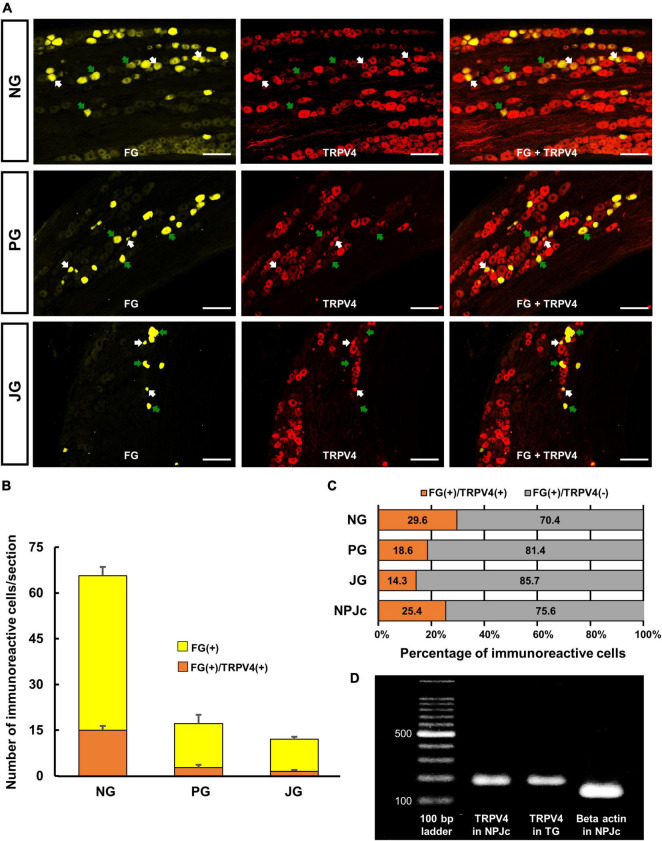
TRPV4 localized on SLN-afferent neurons in the NPJc. **(A)** Expression of TRPV4 in the nodose ganglion (NG), petrosal ganglion (PG), and jugular ganglion (JG). White arrows indicate cells positive for fluorogold (FG) and TRPV4. Green arrows indicate cells positive for FG but negative for TRPV4. Scale bars = 100 μm. **(B)** The number of TRPV4-positive cells/section in the NG, PG, and JG. Data are presented as the mean ± SEM. n = 5. Sections showing the highest number of TRPV4-immunoreactive cells were used for cell counting. Six sections were used from each rat (two sections/ganglion). **(C)** Percentage of FG-stained, TRPV4-positive, or TRPV4-negative cells. FG(+), cells stained with FG; FG(+)/TRPV4(+), FG-stained cells immunopositive for TRPV4; FG(+)/TRPV4(–), FG-stained cells immunonegative for TRPV4. **(D)** *TRPV4* mRNA in NPJc and trigeminal ganglion (TG). TG was used as a positive control.

**FIGURE 3:**
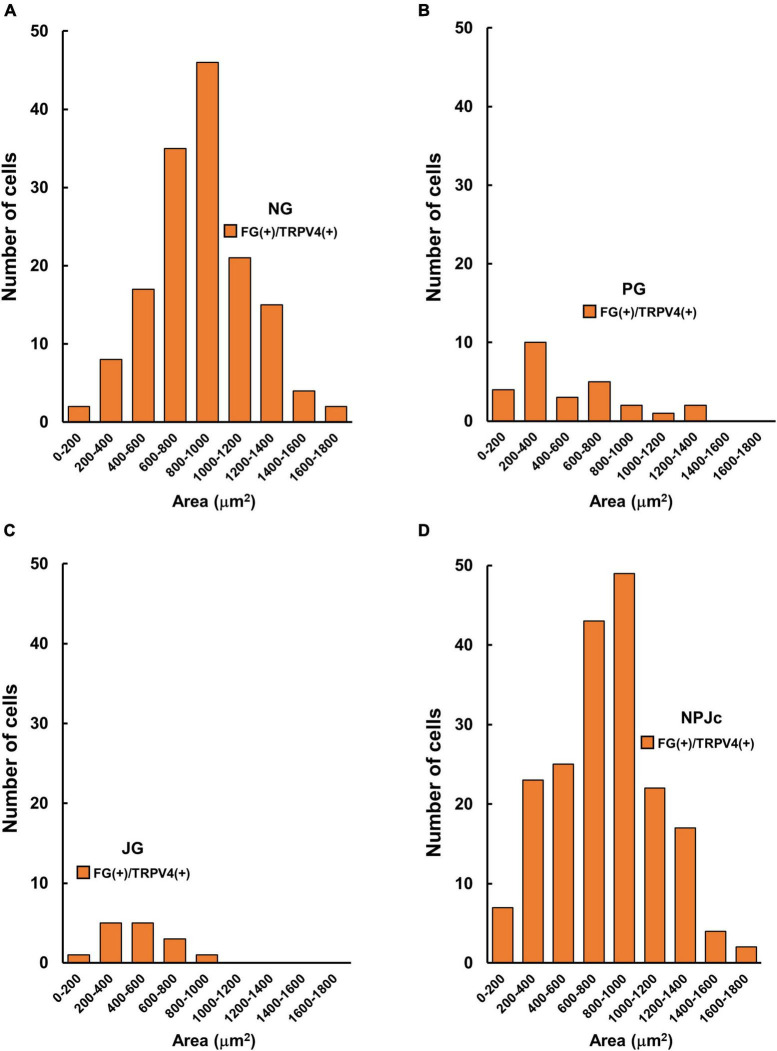
TRPV4-positive cell size in the NPJc. **(A)** Size (area) distribution of TRPV4-positive cells in the nodose ganglion (NG). **(B)** Size (area) distribution of TRPV4-positive cells in the petrosal ganglion (PG). **(C)** Size (area) distribution of TRPV4-positive cells in the jugular ganglion (JG). **(D)** Size (area) distribution of TRPV4-positive cells in the whole NPJc. FG+/TRPV4+, FG-stained cells immunopositive for TRPV4; *n* = 5. Six sections were used from each rat (two sections/ganglion).

We also examined TRPV4 localization in SLN-afferent neurons in the NPJc (Figure 2A). SLN-afferent neurons in the NPJc were traced using FG (a retrograde tracer). Figure 2B shows the number of TRPV4-positive FG-stained SLN-afferent neurons/section of nodose, petrosal, and jugular ganglions. TRPV4 was observed on approximately 25% of the FG-stained SLN-afferent neurons in the NPJc (Figure 2C). We also observed *TRPV4* mRNA in the NPJc using RT-PCR (Figure 2D). Measuring the diameter of FG-stained SLN-afferent neurons in the NPJc (Figure 3) revealed that 49% of the TRPV4-expressing SLN-afferent neurons were large, approximately 35% were medium, and approximately 15% were small in diameter (Figures 3A–D and Table 1).

### 3.3. Topical application of a TRPV4 agonist, dose-dependently facilitated triggering of the swallowing reflex

**FIGURE 4:**
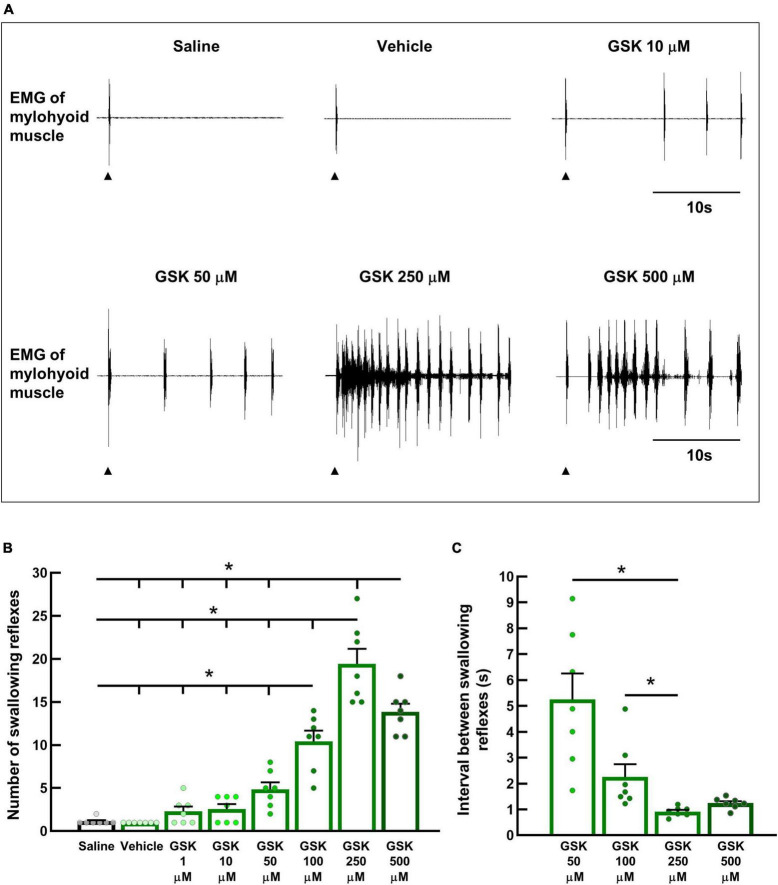
Topical application of GSK1016790A, a TRPV4 agonist, triggered swallowing reflexes in a dose-dependent manner. **(A)** Swallowing reflexes triggered by saline, the vehicle for GSK1016790A, and different GSK1016790A concentrations. Black arrowheads indicate the onset of stimulating solution delivery. **(B)** Comparison of the number of swallowing reflexes triggered by saline, the vehicle for GSK1016790A, and different GSK1016790A concentrations. *P < 0.05 (*F* = 57.34) by one-way repeated measures ANOVA followed by Tukey’s test. **(C)** Comparison of the intervals between the swallowing reflexes triggered by different GSK1016790A concentrations. *P < 0.05 by Friedman repeated measures ANOVA on ranks followed by Tukey’s test. *n* = 7. The number of triggered swallowing reflexes was counted for 20 s following application of the stimulating solutions, and the intervals between the swallowing reflexes were calculated from the reflexes evoked within the 10 s following the onset of solution delivery. Data are presented as the mean ± SEM. Circles in the column graph represent individual data points. S, seconds.

Next, we examined whether TRPV4 in swallowing-related regions can trigger the swallowing reflex. Different concentrations of a potent TRPV4 agonist (GSK1016790A) were topically applied to the SLN-innervated swallowing-related regions. Saline or vehicle for GSK1016790A was also applied to the regions that triggered only one or two swallowing reflexes (Figures 4A, B). GSK1016790A application triggered swallowing reflexes in a dose-dependent manner. The highest number of reflexes were triggered with 250 μM GSK1016790A (19.43 ± 1.75) (Figure 4B), while increasing the GSK1016790A concentration to 500 μM significantly reduced the number of triggered reflexes (13.86 ± 0.94). The number of swallowing reflexes at 100, 250, and 500 μM GSK1016790A was significantly higher than that triggered by 1, 10, 50 μM GSK1016790A, vehicle, and saline (Figure 4B). Additionally, increasing the concentration of GSK1016790A decreased the intervals between the triggered reflexes, and this decrease was most prominent in the initial period following the application of GSK1016790A (Figure 4A). Therefore, we calculated the average interval between reflexes from the initial 10 s period after applying a solution. The mean intervals between the reflexes with 250 μM (0.92 ± 0.07 s) GSK1016790A were significantly shorter than that with 50 μM (5.26 ± 1.00 s), and 100 μM (2.25 ± 0.50 s) GSK1016790A (Figure 4C).

### 3.4. A TRPV4 antagonist significantly reduces the number of GSK1016790A-induced swallowing reflexes

**FIGURE 5:**
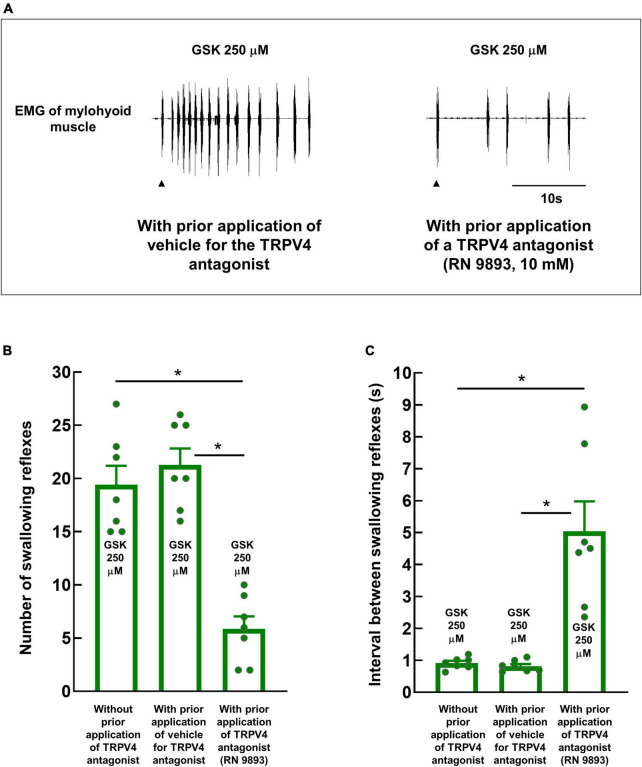
Topical application of RN-9893, a TRPV4 antagonist, prior to the application of GSK1016790A, significantly reduced the number of GSK1016790A-triggered swallowing reflexes. **(A)** Swallowing reflexes triggered by GSK1016790A 250 μM with prior topical application of the TRPV4 antagonist or vehicle. Black arrowheads indicate the onset of stimulating solution delivery. **(B)** Comparison of the numbers of swallowing reflexes triggered by GSK1016790A 250 μM with and without prior application of the TRPV4 antagonist or vehicle. *P < 0.05 (*F* = 34.76) by one-way repeated measures ANOVA followed by Tukey’s test. **(C)** Comparison of intervals between swallowing reflexes triggered by GSK1016790A with and without prior application of the TRPV4 antagonist or vehicle. *P < 0.05 (*F* = 20.38) by one-way repeated measures ANOVA followed by Tukey’s test. *n* = 7. The number of triggered swallowing reflexes was counted for 20 s following application of the stimulating solutions, and the intervals between the swallowing reflexes were calculated from the reflexes evoked within the 10 s following the onset of solution delivery. Data are presented as the mean ± SEM. Circles in the column graph represent individual data points. *P < 0.05 by one-way repeated measures ANOVA followed by Tukey’s test. S, seconds.

The number of swallowing reflexes induced by 250 μM GSK1016790A was significantly reduced by prior topical application of a TRPV4 antagonist, RN-9893 (10 mM) (19.43 ± 1.75 and 5.86 ± 1.18 without and with prior application of the TRPV4 antagonist, respectively) (Figures 5A, B). The vehicle for the antagonist had no significant effect on the number of GSK1016790A-induced swallowing reflexes (21.29 ± 1.54). Additionally, the intervals between the triggered reflexes were significantly increased by the TRPV4 antagonist (0.92 ± 0.07 s and 5.05 ± 0.93 s without and with prior application of RN-9893, respectively) (Figures 5A, C).

### 3.5. Local anesthetic or transection of the bilateral SLNs prior to GSK1016790A application completely abolishes triggering of the swallowing reflex

**FIGURE 6:**
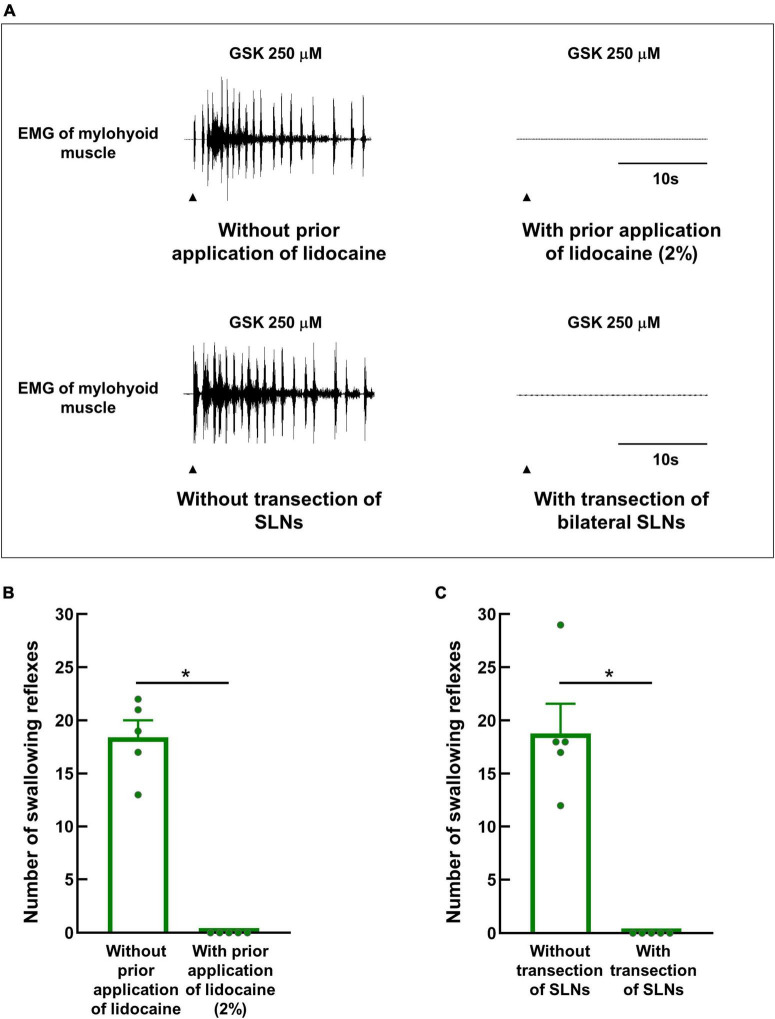
Prior topical application of lidocaine or bilateral transection of SLNs completely prevented the triggering of GSK1016790A-induced swallowing reflexes. **(A)** Swallowing reflexes triggered by GSK1016790A 250 μM with or without prior topical application of lidocaine (2%) or bilateral transection of SLNs. Black arrowheads indicate the onset of stimulating solution delivery. **(B)** Comparison of the numbers of swallowing reflexes triggered by GSK1016790A 250 μM with and without prior application of lidocaine. **(C)** Comparison of the numbers of swallowing reflexes triggered by GSK1016790A 250 μM with and without prior bilateral transection of SLNs. *n* = 5. The number of triggered swallowing reflexes was counted for 20 s following application of the stimulating solutions. Data are presented as the mean ± SEM. Circles in the column graph represent individual data points. *P < 0.05 by paired *t*-test.

To confirm that GSK1016790A triggered swallowing reflexes by excitation of the afferent nerves of the swallowing-related regions, a local anesthetic (2% lidocaine) was applied to the regions 15 minutes prior to GSK1016790A application. Prior lidocaine application completely prevented the triggering of the swallowing reflexes by GSK1016790A (Figures 6A, B).

Additionally, bilateral SLNs were transected prior to the application of GSK1016790A to confirm that the swallowing reflexes triggered by GSK1016790A involved the SLN afferents but not the spinal nerve afferents. GSK1016790A could not trigger swallowing reflexes following transection of the SLNs (Figures 6A, C).

## 4. Discussion

To the best of our knowledge, this study is the first to report the functional involvement of TRPV4 in triggering the swallowing reflex. TRPV4 is expressed in various structures, including the epithelial cells of the choroid plexus (Takayama et al., 2014), skin keratinocytes (Sokabe et al., 2010), endothelial cells of blood vessels (Hatano et al., 2013), astrocytes (Benfenati et al., 2011), epithelial cells of the trachea (Lorenzo et al., 2008), larynx (Hamamoto et al., 2008; Foote et al., 2022), lungs (Fernández-Fernández et al., 2008), cornea (Mergler et al., 2011), esophagus (Mihara et al., 2011; Ueda et al., 2011), stomach (Mihara et al., 2016), bladder (Birder et al., 2007; Janssen et al., 2011), oral mucosa (Nakatsuka and Iwai, 2009; Moayedi et al., 2022; Yahya et al., 2022), odontoblasts (Egbuniwe et al., 2014), taste buds (Matsumoto et al., 2019), hippocampal neurons (Shibasaki et al., 2007), dorsal root ganglia (Cao et al., 2009; Liu et al., 2010; Lechner et al., 2011), trigeminal (Denadai-Souza et al., 2012; Chen et al., 2013, 2014, 2020), and vagal ganglia (Bonvini et al., 2016), and nerve fibers in the dental pulp (Bakri et al., 2018). Here we report TRPV4 on nerve fibers in SLN-innervated swallowing-related regions. TRPV4 was also localized on cell bodies of SLN-afferent neurons located in the NPJc, with the majority on large to medium-diameter neurons. We also observed that topical application of a potent TRPV4 agonist into the SLN-innervated swallowing-related regions dose-dependently triggered swallowing reflexes. The number of agonist-induced reflexes was significantly attenuated by prior topical application of a TRPV4 antagonist. The above observations indicate that activation of TRPV4 expressed on sensory nerves of the peripheral swallowing-related regions excites the nerves leading to activation of the sCPG to trigger the swallowing reflex. In addition to nerves, we observed TRPV4 on some epithelial cells, cells of taste-bud-like structures, endothelial cells, and seromucous glands. TRPV4 present in these structures may also contribute to triggering the swallowing reflex, although the mechanism of this action is not known. One possibility is that activation of TRPV4 in these structures, especially on epithelial cells, may release adenosine triphosphate (ATP), which can excite adjacent nerve fibers that contribute to triggering the swallowing reflex. In this context, ATP is released from gastric and esophageal epithelial cells by the TRPV4 agonist, GSK1016790A (Mihara et al., 2011, 2016).

Our findings of TRPV4 localization in the peripheral swallowing-related regions and its involvement in triggering the swallowing reflex have clinical significance. The results may indicate TRPV4 as a target to develop a pharmacological treatment for the management of oropharyngeal dysphagia. Various TRP channels are expressed on the nerves of the swallowing-related regions (Yamamoto et al., 2007; Alvarez-Berdugo et al., 2016; Hossain et al., 2018, 2020a,b,^2022^). Additionally, activation of TRPV1, TRPA1, and TRPM8 present in the swallowing-related regions triggers the swallowing reflex (Hossain et al., 2018, 2020a,^2022^). Furthermore, in clinical studies, TRPV1, TRPA1, and TRPM8 agonists improved the efficacy, safety, and physiology of swallowing in patients with oropharyngeal dysphagia (Ebihara et al., 1993, 2006; Rofes et al., 2013, 2014; Ortega et al., 2016; Shin et al., 2016; Nakato et al., 2017; Alvarez-Berdugo et al., 2018; Tomsen et al., 2019, 2020, 2022; Cheng et al., 2022). Therefore, TRP channels have emerged as potential pharmacological targets for developing treatments to manage oropharyngeal dysphagia (Hossain et al., 2020a; Cheng et al., 2022; Tomsen et al., 2022). Clinical guidelines for the management of neurogenic dysphagia recommend TRP channel agonists as pharmacological therapies with behavioral swallowing interventions for patients with a delay in their swallowing response (Dziewas et al., 2021a,b).

Our findings also provide insight into the physiological significance of TRPV4 in triggering the swallowing reflex. Triggering the swallowing reflex by applying a TRPV4 agonist indicates that TRPV4 functions as a chemosensor to trigger the swallowing reflex. TRPV4 is a polymodal channel and can therefore be activated by various stimuli, including chemical, mechanical, osmotic, acidic pH, and temperature stimuli (Liedtke et al., 2000; Strotmann et al., 2000; White et al., 2016; Rosenbaum et al., 2020). We and others have reported triggering of the swallowing reflex by mechanical stimuli (Kitagawa et al., 2002; Tsujimura et al., 2019; Hossain et al., 2022). Additionally, hypoosmotic stimuli (e.g., water) and weak acidic stimuli can induce the swallowing reflex (Shingai et al., 1989; Kajii et al., 2002; Hossain et al., 2018, 2020a). Therefore, in addition to acting as a chemosensor, TRPV4 in the swallowing-related regions has the potential to act as another sensor (e.g., mechanosensor or osmosensor) to trigger the swallowing reflex. Future studies may reveal its involvement in other-stimuli-induced swallowing reflexes. In this context, we observed TRPV4 localization on neurons of various diameters in the NPJc (Table 1). Variability in the diameters of TRPV4-positive neurons may indicate their variability in functions. For example, large-diameter TRPV4-positive neurons may be involved in mechanical stimuli-induced swallowing reflex.

We observed that topical application of a local anesthetic to the swallowing-related regions completely abolished triggering of swallowing reflex by the TRPV4 agonist confirming the involvement of sensory nerves in triggering the swallowing reflex. To focus on SLNs, we recorded the reflexes with intact SLNs, but with the other nerves (bilateral IX-ph, X-ph, IX-li, and RLN) transected because they may carry sensory information to the sCPG to trigger the swallowing reflex. Some branches of the spinal nerves may also innervate the swallowing-related regions, although the sensory information carried through the spinal nerves may not play a significant role in triggering the swallowing reflex. Sensory information carried through the vagus, glossopharyngeal, trigeminal, and facial nerves are pivotal in triggering the swallowing reflex (Yoshida et al., 2000; Jean, 2001; Kitagawa et al., 2002; Yamamura et al., 2010; Hossain et al., 2020a). We confirmed the involvement of SLNs in carrying sensory information for triggering the swallowing reflex under our experimental conditions in an additional experiment. We transected the bilateral SLNs (Figure 6), which completely abolished triggering of the swallowing reflex by the TRPV4 agonist. This confirmed involvement of the SLN-afferents but not the spinal afferents in triggering the swallowing reflex under the experimental conditions of the study.

In conclusion, our findings reveal that TRPV4 is present on the afferent nerves innervating the peripheral swallowing-related regions and is functional in triggering the swallowing reflex. TRPV4 can be pharmacologically targeted to facilitate swallowing to manage oropharyngeal dysphagia.

## Data availability statement

The raw data supporting the conclusions of this article will be made available by the authors, without undue reservation.

## Ethics statement

This animal study was reviewed and approved by Matsumoto Dental University Intramural Animal Care and Veterinary Science Committee, Matsumoto Dental University, Shiojiri, Japan.

## Author contributions

MH and JK: conception and design of the work. All authors: acquisition, analysis, or interpretation of data and drafting the manuscript or revising it critically.
