# Supplementary material for: Pharmacological activation of transient receptor potential vanilloid 4 promotes triggering of the swallowing reflex in rats
Source: Front Cell Neurosci. 2023 Feb 22;17:1149793. doi: 10.3389/fncel.2023.1149793 (PMC9992545; doi:10.3389/fncel.2023.1149793)
Supplement: Supplementary file 1 [file Data_Sheet_1.docx]

Supplementary Material

# Supplementary Figures


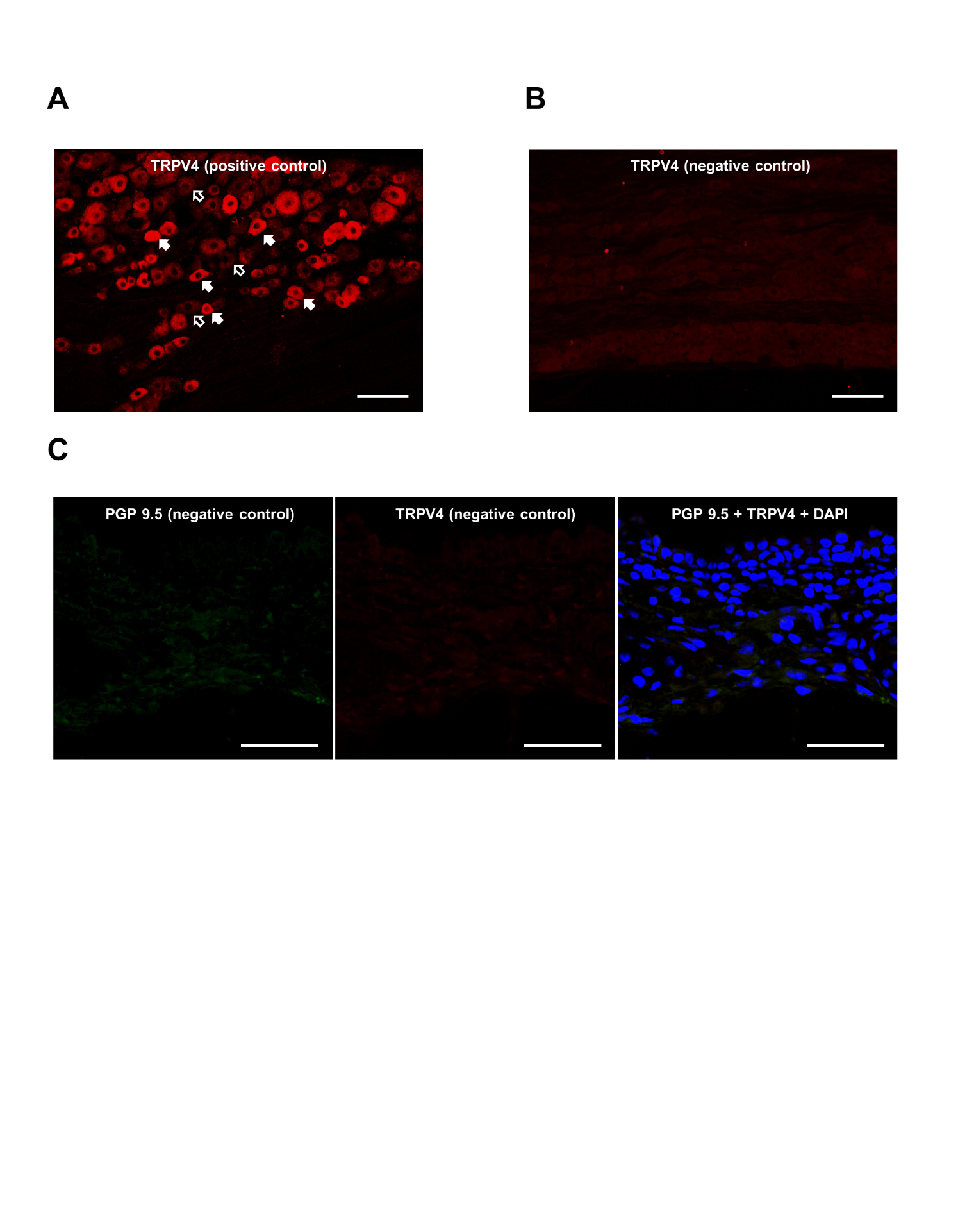


**Supplementary Figure 1.** **Positive and negative controls for the TRPV4 antibody**. (A) Representative photomicrograph of a rat trigeminal ganglion section that was used as a positive control for the TRPV4 antibody (rabbit monoclonal anti-TRPV4 antibody; Cat# ab259361; Abcam, Cambridge, UK). White arrows indicate examples of cells positive for TRPV4. Black arrows indicate examples of cells negative for TRPV4. Scale bar = 100 μm. (B) Representative photomicrograph of a nodose ganglion section that was used as a negative control for the anti-TRPV4 antibody. Scale bar = 100 μm. A universal negative control reagent containing a mixture of purified rabbit, mouse, and goat immunoglobulin was used instead of the primary antibody. (C) Representative photomicrographs of a section taken from the SLN-innervated region used as a negative control for the anti-TRPV4 and anti-PGP 9.5 primary antibodies. DAPI was used to visualize cell nuclei. Scale bars = 50 μm. A universal negative control reagent containing a mixture of purified rabbit, mouse, and goat immunoglobulin was used instead of the primary antibodies.
